# Mesenchymal stem cells and cutaneous wound healing: novel methods to increase cell delivery and therapeutic efficacy

**DOI:** 10.1186/s13287-016-0303-6

**Published:** 2016-03-09

**Authors:** Dylan E. Lee, Nagi Ayoub, Devendra K. Agrawal

**Affiliations:** Department of Clinical & Translational Science, Creighton University School of Medicine, CRISS II Room 510, Omaha, NE 68178 USA

## Abstract

Mesenchymal stem cells (MSCs) (also known as multipotent mesenchymal stromal cells) possess the capacity for self-renewal and multi-lineage differentiation, and their ability to enhance cutaneous wound healing has been well characterized. Acting via paracrine interactions, MSCs accelerate wound closure, increase angiogenesis, promote resolution of wound inflammation, favorably regulate extracellular matrix remodeling, and encourage regeneration of skin with normal architecture and function. A number of studies have employed novel methods to amplify the delivery and efficacy of MSCs. Non-traditional sources of MSCs, including Wharton’s jelly and medical waste material, have shown efficacy comparable to that of traditional sources, such as bone marrow and adipose tissue. The potential of alternative methods to both introduce MSCs into wounds and increase migration of MSCs into wound areas has also been demonstrated. Taking advantage of the associations between MSCs with M2 macrophages and microRNA, methods to enhance the immunomodulatory capacity of MSCs have shown success. New measures to enhance angiogenic capabilities have also exhibited effectiveness, often demonstrated by increased levels of proangiogenic vascular endothelial growth factor. Finally, hypoxia has been shown to have strong wound-healing potential in terms of increasing MSC efficacy. We have critically reviewed the results of the novel studies that show promise for the continued development of MSC-based wound-healing therapies and provide direction for continued research in this field.

## Background

Mesenchymal stem cells (MSCs) are characterized by the ability to self-renew and exhibit differentiation into multiple tissue-forming cell lineages, such as osteoblasts, adipocytes, chondrocytes, tenocytes, and myocytes [1]. Obtainable from a number of tissues, including bone marrow, adipose tissue, and the umbilical cord, MSCs are also characterized by the expression of surface CD markers, including CD44^+^, CD73^+^, CD90^+^, and CD105^+^, and are distinguished from hematopoietic cells by a lack of CD34, CD45, CD14, and HLA-DR [1]. MSCs have also been shown to exhibit immunomodulatory, reparative, and regenerative effects through paracrine signaling, suggesting great therapeutic potential [2, 3].

Wound healing is a complex process requiring cell migration, inflammation, angiogenesis, granulation tissue formation, re-epithelialization, and extracellular matrix (ECM) remodeling. MSCs have an active role through this process, and therapeutic application of MSCs has been shown to enhance and improve wound-healing outcomes. Here, we critically reviewed the ability of MSCs to enhance cutaneous wound healing and discussed the novel methods used to increase MSC delivery and efficacy.

## Mesenchymal stem cells enhance wound healing

**Fig. 1:**
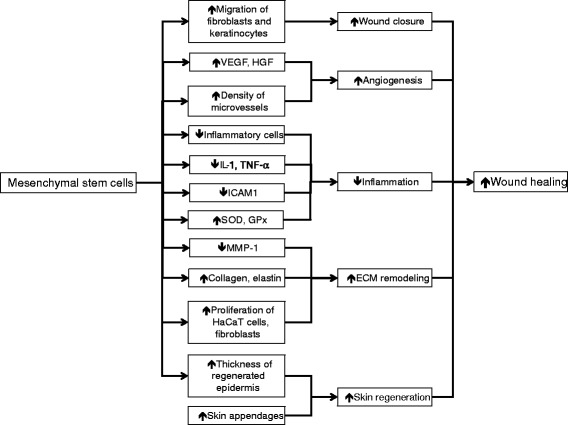
Enhanced cutaneous wound healing by mesenchymal stem cells. This involves accelerating wound closure, increasing angiogenesis, decreasing wound inflammation, positively regulating extracellular matrix (*ECM*) remodeling, and promoting regeneration of normal skin architecture and functioning. These effects are mediated via paracrine signaling. *GPx* glutathione peroxidase, *HaCaT* immortalized human keratinocyte, *HGF* hepatocyte growth factor, *ICAM1* intercellular adhesion molecule 1, *IL-1* interleukin-1, *MMP-1* matrix metalloproteinase-1, *SOD* superoxide dismutase, *TNF-α* tumor necrosis factor-alpha, *VEGF* vascular endothelial growth factor

By accelerating wound closure, enhancing re-epithelialization, increasing angiogenesis, promoting granulation tissue formation, modulating inflammation, and regulating ECM remodeling, administration of MSCs has demonstrated a beneficial effect on cutaneous wound healing and skin regeneration (Fig. 1). Importantly, this beneficial effect appears to be mediated by paracrine signaling [2, 3].

### Wound closure

Multiple studies have shown that MSCs enhance wound healing by accelerating wound closure [4–13]. Walter et al. [4] demonstrated that human bone marrow-derived MSCs increased wound closure rate by increasing the in vitro migration of fibroblasts and keratinocytes. Similar findings were reported by Smith et al. [5], who found that the accelerated wound closure in the presence of murine bone marrow-derived MSCs was due to increased dermal fibroblast migration, and Jeon et al. [6], who demonstrated that human skin fibroblasts cultured with human umbilical cord blood MSC-conditioned media exhibited significantly elevated migratory ability.

### Angiogenesis

MSCs enhance angiogenesis during the wound-healing process. For example, treatment of rat wound with human umbilical cord MSCs increases the levels of vascular endothelial growth factor (VEGF) (one of the most potent pro-angiogenic factors), the density of microvessels, and cutaneous wound microcirculation [8]. Data from a study of rat adipose-derived stem cells implanted into rat wounds suggested that the MSCs could participate in vasculogenesis of wound healing through direct differentiation into vascular endothelial cells [12], a finding consistent with other studies [14, 15]. These same MSCs also secreted significantly higher levels of the pro-angiogenic cytokines VEGF and hepatocyte growth factor [12]. In another study, Schlosser et al. [3] demonstrated that injected murine bone marrow MSCs reduced arteriolar vascular resistance and increased functional capillary density in the vasculature of murine skin recovering from ischemia. The key finding in this study was the expression of pro-angiogenic cytokines such as VEGF by the MSCs.

### Immunomodulation

Resolution of inflammation is essential to successful wound healing, and chronic inflammation can lead to poor healing outcomes [16]. The ability of MSCs to modulate the inflammatory response in wounds supports their favorable effect on the healing response. Cutaneous rat wounds transplanted with human umbilical cord MSCs demonstrated a significantly lower number of inflammatory cells and pro-inflammatory cytokines such as interleukin (IL)-1 and tumor necrosis factor-alpha (TNF-α) [8]. Importantly, these wounds also exhibited a shorter recovery time and faster healing rate. Smith et al. [5] demonstrated that human dermal fibroblasts co-cultured with murine bone marrow-derived MSCs had decreased mRNA levels of intercellular adhesion molecule 1 (ICAM1). Because it has been postulated that ICAM1 mediates binding of leukocytes to dermal fibroblasts, indicating a role in inflammation, downregulation of ICAM1 expression by these fibroblasts may be necessary for resolution of inflammation during wound repair [17]. A study by Jeon et al. [6] evaluating the antioxidant activity of fibroblasts exposed to human umbilical cord MSC-conditioned media demonstrated that the conditioned media significantly promoted superoxide dismutase and glutathione peroxidase (GPx) synthesis. This detoxifying ability is significant as superoxide detoxification at wound sites promotes proper wound healing [18]. Moreover, GPx-mediated detoxification of hydrogen peroxide has been shown to be important for upregulation of VEGF in skin wounds, suggesting a pro-angiogenic effect [6].

### Extracellular matrix remodeling

MSCs have also exhibited an ability to enhance proper ECM events during the healing process [2, 6, 19]. Conditioned media from human umbilical cord blood MSCs have been shown to inhibit the expression of matrix metalloproteinase (MMP)-1, which suggests that MSCs suppress degradation of collagenous matrix and serve to preserve the matrix and contribute to fibroblast regeneration [6]. In the same study, this media also significantly increased the production of collagen and elastin by fibroblasts, further suggesting an enhancement of wound healing. Using human adipose-derived MSCs and an in vitro wound-healing assay, Lee et al. [2] demonstrated that treatment with MSCs significantly increased the proliferation of HaCaT (immortalized human keratinocyte) cells and dermal fibroblasts and increased overall wound healing. These MSCs also enhanced the ability of dermal fibroblasts to contract collagen lattices, implying that the MSCs induce fibroblasts to transform into myofibroblasts.

### Skin regeneration

The optimal outcome of cutaneous wound healing involves regeneration of the normal architecture and function of the skin. Several studies have shown that MSCs are capable of such regenerative capacity [12, 17, 19, 20]. Luo et al. [20], examining the effect of human umbilical cord blood MSCs on severe combined immunodeficient (SCID) mice, reported that, in addition to significantly enhancing wound-healing rate, MSCs increased the thickness of the regenerated epidermis, increased the dermal ridges and amount of cells in regenerated skin, and produced healing tissue with more regular alignment of fibers. Additionally, the MSC-treated wounds developed hair follicles, sweat glands, and other normal skin appendages. These results suggest that the administration of MSCs not only may be able to accelerate wound healing but also could enhance wound-healing quality and the physiological functioning of the regenerated skin. Another study using an in vitro human keratinocyte-MSC skin model demonstrated that skin models in which keratinocytes were cultured with MSCs gave rise to a multi-layered, well-differentiated epidermis [19]. These models also expressed collagen, indicating the ability of MSCs to regulate ECM production.

## Novel methods to increase mesenchymal stem cell delivery and efficacy

The strong therapeutic potential of MSCs in cutaneous wound healing has led to efforts to determine the optimal sources and delivery methods for MSCs. Recent studies have also evaluated the ability of novel methods to maximize the migratory, immunomodulatory, angiogenic, and reparative abilities of MSCs (Table 1).

**Table 1:** Summary of the key findings on the therapeutic effects of MSCs

Therapeutic effect on MSCs	Method/finding	Source of MSCs	Wound-healing model	Reference
Increased delivery	Three-dimensional collagen allograft	Rat bone marrow	Rat	[29]
	Microsphere-based engineered skin loaded with EGF	Mouse bone marrow	Mouse	[31]
Increased migration	CXCL12/CXCR4 axis	Mouse bone marrow	Mouse	[32]
	Platelet-rich plasma	Human amniotic fluid	Human	[34]
	EMPB	Mouse	Mouse	[35]
Increased survival	Biomimetic pullulan-based hydrogel	Mouse	Mouse	[36]
Increased immunomodulation	Polarization of macrophages to M2 phenotype	Human gingival tissue	Mouse	[37]
	Increased miRNA-146a	Mouse bone marrow	Mouse	[16]
	MSC-derived TSG-6	Human bone marrow	Mouse	[42]
Increased angiogenesis	Biomimetic hydrogel scaffold	Mouse bone marrow	Mouse	[43]
	LLLT	Dog adipose tissue	Mouse	[44]
	14*S*,21*R*-diHDHA	Mouse	Mouse	[45]
	v-myc introduction	Human adipose tissue	Mouse	[47]
Increased wound-healing efficacy	Hypoxia	Human amniotic fluid	Mouse	[49]
	Hypoxia	Human adipose tissue	Mouse	[50]
	Hypoxia	Human bone marrow	Mouse	[51]

*14S,21R-diHDHA* 14*S*,21*R*-dihydroxydocosa-4*Z*,7*Z*,10*Z*,12*E*,16*Z*,19*Z*-hexaenoic acid, *EGF* epidermal growth factor, *EMPB Mallotus philippinensis* bark, *LLLT* low-level laser therapy, *miRNA* microRNA, *MSC* mesenchymal stem cell, *TSG-6* tumor necrosis factor-alpha-stimulated protein 6

### Sources for mesenchymal stem cells

**Fig. 2:**
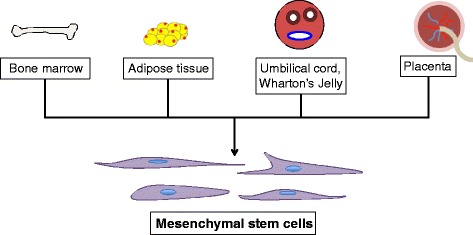
Sources of mesenchymal stem cells. Mesenchymal stem cells can be obtained from various sources, including bone marrow, adipose tissue, the umbilical cord, Wharton’s jelly, and the placenta

Although bone marrow and adipose tissue have proven to be sources for effective MSCs, several disadvantages exist, especially for bone marrow MSCs. These include availability, ethical concerns, and invasive and painful procedures required for obtainment [21]. As a result, alternative sources for MSCs have been considered and analyzed (Fig. 2). A study by Arno et al. [22] found that Wharton’s jelly-derived MSCs, which had not yet been studied in the context of human skin, caused normal human skin fibroblasts to coapt wound borders faster in vitro. More significantly, mice treated with these MSCs showed increased wound-healing rates, increased re-epithelialization, and more organized ECM, suggesting that Wharton’s jelly-derived MSCs promoted wound healing in vivo. In addition, these MSCs upregulated gene expression of wound-healing factors, including transforming growth factor-beta (TGF-β), hypoxia-inducible factor-1α, and plasminogen activator inhibitor-1. Azari et al. [23] similarly found that Wharton’s jelly MSCs from caprine umbilical cords enhanced re-epithelialization of caprine cutaneous wounds. Treated wounds also exhibited less inflammatory cell infiltration.

MSCs obtained from medical waste material provide a number of advantages, including abundant availability and general absence of ethical concerns. The effect of human placenta-derived MSCs on the wound-healing rate was evaluated in diabetic Goto-Kakizaki rats, and it was found that placenta-derived MSC treatment resulted in significantly smaller wounds with increased collagen deposition, thicker granulation tissue, thicker epidermal layers, more regular alignment of fibers, and greater neovasculariztion [21]. These results demonstrated for the first time that placenta-derived MSCs, which are readily available from maternity wards, remarkably increased wound-healing rates. In another study, adipose-derived MSCs from the hypodermis of discarded skin samples from severely burned patients were demonstrated to maintain their stemness and were postulated to have contributed significantly to the wound healing and regenerative process [24].

The efficacy of MSCs from different sources appears to vary, and comparisons have accordingly been performed. A study by Hsieh et al. [25] comparing the angiogenic capabilities of Wharton’s jelly MSCs and bone marrow MSCs found that more human microvascular endothelial cells moved toward Wharton’s jelly MSCs compared with bone marrow MSCs. The total vessel length generated by these cells was also longer when incubated with Wharton’s jelly MSC-conditioned medium compared with bone marrow MSC-conditioned medium. Evaluating diabetic murine skin wounds, Kim et al. [13] determined that, compared with human adipose-derived MSCs, human amniotic MSCs significantly increased the rate of fibroblast wound closure. These wounds also displayed enhanced re-epithelialization compared with wounds treated with adipose-derived MSCs. Contrasting results were seen by Liu et al. [26], who reported that human adipose MSCs, compared with human amnion MSCs and human bone marrow MSCs, had the most pronounced effect on murine wound closure and were associated with the greatest re-epithelialization and thickest granulation tissue. The adipose-derived MSCs also had the greatest effect on human dermal fibroblast migration and expression of type I collagen. Moreover, fibroblasts cultured with adipose MSCs had significantly higher expression of VEGF, basic fibroblast growth factor (bFGF), and TGF-β. The discrepancy between these results suggests a need for future investigation into which source provides MSCs that exhibit the greater improvement in wound healing and therapeutic efficacy.

### Delivery methods

Traditionally, most studies have used the technique of injecting MSCs intradermally into or around the wound area. Although this method has been shown to improve wound healing, the ultimate therapeutic potential of MSCs appears to be limited because of poor engraftment efficiency and cell retention at the wound site [27]. Moreover, the lack of sufficient MSC engraftment at sites of injury is cited as a major limiting factor of current MSC-based therapies [28]. Alternative methods that increase the delivery or survival (or both) of MSCs at the wound site are henceforth desirable. Kim et al. [29] employed a three-dimensional collagen allograft model, which is most similar to the skin dermis structure, to apply rat MSCs to wounds. This resulted in increased neovascularization, accelerated healing, and, interestingly, increased early expression of MMP-9, which is thought to play a critical role in degrading ECM to liberate VEGF and facilitate the initial steps of angiogenesis. MMP-9 has also been reported to stimulate the production and secretion of VEGF in retinal pigment epithelial cells [30]. Huang et al. [31] alternatively used microsphere-based engineered skin containing murine bone marrow-derived MSCs loaded with epidermal growth factor (EGF) and evaluated its effect on murine wound healing. In these studies, a higher rate of wound repair was found with thicker granulation tissue, increased vasculature, and increased regeneration of sweat gland-like structures compared with engineered skin not loaded with EGF. This novel delivery system highlights the therapeutic potential of adding EGF to MSC delivery templates.

Investigators have also explored ways to increase MSC mobilization and migration into cutaneous wounds. A study examining the role of CXCL12/CXCR4 signaling in the migration of murine bone marrow-derived MSCs determined that the expression of CXCL12 (also known as stromal derived factor-1) and CXCR4 was significantly elevated in murine wounds treated with bone marrow-derived MSCs [32]. Perhaps more importantly, an antagonist of CXCR4 inhibited the directional migration of bone marrow MSCs in vitro, thus preventing MSCs from participating in wound repair. These results coincided with those of other studies, which found that CXCL12 induced migration of MSCs following binding to its cognate receptor, CXCR4 [33], and point to the CXCL12/CXCR4 axis as a target for novel therapeutic strategies. Similar migratory findings were observed in a study by Roubelakis et al. [34], who reported that platelet-rich plasma, a rich source of growth factors, significantly increased the migration ability of human amniotic fluid MSCs and accelerated the healing process. Using *Mallotus philippinensis* bark (EMPB) extract, Furumoto et al. [35] likewise demonstrated an enhancement of murine MSC migration, both into the blood circulation and to the wound site. Application of EMPB, the main activity of which is mediated by cinnamtannin B-1, further resulted in improved wound healing and significant increases in capillary number, suggesting a pro-angiogenic role.

Prolonging the survival of MSCs in wounds would also be expected to improve healing outcomes. Murine MSCs seeded in a biomimetic hydrogel based on the carbohydrate pullulan, an alpha-(1–4)- and alpha-(1–6)-glucan produced by the fungus *Aureobasidium pullulans*, was seen to act as an antioxidant scaffold that improved MSC survival by potentially protecting MSCs from high levels of free radicals (which can impair MSC survival) [36]. This finding agrees with the aforementioned idea that detoxification of free radicals at wound sites promotes proper wound healing [18].

### Enhanced immunomodulation

Enhancing the immunomodulatory abilities of MSCs would theoretically lead to better resolution of wound inflammation. A study by Zhang et al. [37] found that macrophages co-cultured with human gingival tissue MSCs acquired the anti-inflammatory M2 phenotype, demonstrated by increased expression of CD206 (a well-accepted marker for the M2 macrophage) [38–40], a high level of the anti-inflammatory cytokine IL-10, and a low level of the pro-inflammatory cytokine TNF-α. Importantly, injection of the gingival MSCs also resulted in attenuated local inflammation, increased angiogenesis, and significantly accelerated murine wound closure, suggesting that gingival MSCs can promote cutaneous wound repair by eliciting the polarization of macrophages toward an M2 phenotype. These results corroborate the findings of other studies showing that M2 macrophages, also known as “wound-healing macrophages”, produce mediators essential for tissue remodeling and the resolution of inflammation, thus promoting wound repair [38, 41].

Another study evaluated the relationship between microRNA and MSCs in wound healing [16]. MicroRNAs negatively regulate gene expression, and miRNA-146a has specifically been reported to negatively regulate the innate immune response. Evidence for a role of miRNA-146a in wounds was seen in a diabetic murine wound-healing model revealing significantly reduced miRNA-146a expression levels and a dramatic increase in pro-inflammatory genes. Treatment of these wounds with murine bone marrow MSCs resulted in improved wound closure, significantly increased miRNA-146a expression, and downregulation of the pro-inflammatory genes. These results suggest that modulation of miRNA-146a levels by MSC treatment can decrease inflammation and enhance wound closure; miRNA-146a may also be a future therapeutic target for MSC-based wound therapies [16].

Further insight into the immunomodulatory capacity of MSCs was provided by Qi et al., who found that human bone marrow MSC-derived TNF-α-stimulated protein 6 (TSG-6) was shown to suppress macrophage TNF-α release, indirectly contributing to accelerated murine wound healing [42]. These results were confirmed by the fact that TSG-6-silenced MSCs failed to suppress macrophage-derived TNF-α release. Thus, MSCs may contribute to resolution of inflammation, and consequently promote wound healing, by inhibiting release of the pro-inflammatory cytokine TNF-α. MSC-derived TSG-6 may have a role in the development of MSC-based wound therapies.

A mechanism by which MSCs exert immunomodulatory effects has been proposed by Waterman et al., who reported that stimulation of Toll-like receptor (TLR)3 of human MSCs resulted in polarization toward an immunosuppressive phenotype (MSC2) needed for anti-inflammatory responses and tissue healing [43]. Investigators also noted that stimulation of TLR4 of MSCs contrastingly resulted in polarization toward a pro-inflammatory phenotype (MSC1) theorized to be important for early response to tissue injury. It was therefore suggested that the default MSC phenotype is immunosuppressive (to prevent detrimental effects from a pro-inflammatory one) and that MSCs can become pro-inflammatory to promote early tissue repair. The role of TLR3 and TLR4 stimulation in the immunomodulatory capabilities of MSCs henceforth deserves further investigation, and studies examining this mechanism in cutaneous wound healing may provide avenues for MSC-based wound treatments.

### Enhancement of angiogenesis

The pro-angiogenic role of MSCs has been well demonstrated by multiple studies. Efforts to maximize this capacity would be expected to increase the beneficial role of MSCs on wound healing. Employing a biomimetic hydrogel scaffold to deliver murine bone marrow-derived MSCs into murine wounds, Rustad et al. noted that MSCs cultured within the hydrogels significantly increased VEGF levels, brought about a faster rate of wound healing, resulting in more improved skin architecture (with greater return of hair follicles and sebaceous glands) compared with local injection of MSCs [44]. Additionally, investigators observed a moderate increase in microvessel number in wounds treated with injected MSCs, but noted that neovascularization was further augmented when MSCs were delivered within the hydrogel scaffold. Interestingly, levels of MMP-9, which was previously mentioned to have a potential pro-angiogenic role, were also significantly higher in wounds treated with MSC-seeded scaffolds. In a separate study, Kim et al. [45] demonstrated that low-level laser therapy (LLLT) amplified the angiogenic ability of canine adipose-derived MSCs. This was demonstrated by significantly increased numbers of vasculature structures and greater levels of MSC-derived VEGF and bFGF in murine wounds treated with MSCs and LLLT compared with wounds treated solely with MSCs. The group treated with MSCs and LLLT also showed a significantly smaller wound area, increased numbers of hair follicles and sebaceous glands, and a decreased percentage of caspase 3-positive MSCs (with a corresponding increase in the number of MSCs), suggesting a pro-survival, anti-apoptotic role. LLLT may therefore enhance efficacy and survival of MSCs.

In another study, Tian et al. demonstrated that 14*S*,21*R*-dihydroxydocosa-4*Z*,7*Z*,10*Z*,12*E*,16*Z*,19*Z*-hexaenoic acid (14*S*,21*R*-diHDHA) administration induced VEGF secretion by murine MSCs [46]. The 14*S*,21*R*-diHDHA is a novel endogenous lipid mediator derived from docosahexaenoic acid that has recently been shown to be capable of promoting wound healing and angiogenesis [47]. Together, 14*S*,21*R*-diHDHA and MSCs appeared to act synergistically to promote wound angiogenesis [46]. The 14*S*,21*R*-diHDHA also enhanced the paracrine function of MSCs, leading to significantly increased murine wound healing, accelerated re-epithelialization, and promoted granulation tissue and vasculature formation. Notably, skin wounds of diabetic mice exhibited decreased formation of 14*S*,21*R*-diHDHA; thus, 14*S*,21*R*-diHDHA may serve as a basis for the development of treatment strategies for chronic wounds [46].

Genetic modification of MSCs has also been explored. A study by Song et al. [48] found that introduction of v-myc into human adipose-derived MSCs using a lentiviral gene transfer system resulted in increased MSC secretion of VEGF and increased vessel formation. Investigators also took advantage of Akt1, the expression of which increases MSC survival and tissue repair ability [49]. Constitutively active phosphorylation in Akt1 further increased VEGF secretion by v-myc MSCs and also caused accelerated murine wound closure, decreased wound inflammation, and improved epidermis formation [48].

### Efficacy

Several studies have shown that hypoxia can enhance the efficacy of MSCs in regard to cutaneous wound healing. Using human amniotic fluid MSCs in a murine wound-healing model, Jun et al. [50] found that MSCs cultured in hypoxic conditions, compared with MSCs cultured in normoxic conditions, had increased viability and proliferation as well as increased expression of VEGF. These MSCs also increased the viability and migration rate of human dermal fibroblasts, increased expression of ECM molecules (such as type III collagen, fibronectin, and elastin), and accelerated wound closure. Similar results were reported by Lee et al. [51], Kong et al. [21], and Chen et al. [52], who evaluated the effect of hypoxia on human adipose-derived MSCs, human placenta-derived MSCs, and human bone marrow-derived MSCs, respectively. Thus, hypoxia may have great therapeutic potential in terms of enhancing the ability of MSCs to promote proper wound healing and regenerative skin repair.

## Conclusions

The beneficial effect of MSCs on cutaneous wound healing has been well established. Through paracrine interactions, MSCs increase wound closure rates, promote angiogenesis, decrease wound inflammation, regulate ECM healing events, and enhance regeneration of proper skin structure and function. Recent studies have evaluated novel methods to increase the therapeutic capability of MSCs. These measures have been shown to increase MSC migration and survival, enhance immunomodulation, promote angiogenic capacity, and improve overall efficacy. These results show promise for the further development of MSC-based therapeutic strategies. Future studies can further establish this research and provide insight into how these findings can be applied in the clinical arena. More work is also needed to determine the ideal source of MSCs, taking into account therapeutic efficacy as well as availability, ethical issues, and necessary procedures.

The therapeutic effects discussed in this review also support a potential role for MSCs in recognized clinical wound treatment models. For example, the wound bed preparation model published by Sibbald et al. recognizes prolonged inflammation as a principal component of local wound care that requires clinical optimization [53]. This model discusses the fact that inflammation contributes to total wound pain and is a driving factor in the persistence of chronic wounds (diabetic foot ulcers, pressure ulcers, venous ulcers, and so on), which represent a substantial burden on a patient’s activities of daily living as well as health-care systems worldwide. Because of their immunomodulatory and angiogenic properties, MSCs could be inserted into this model. A therapeutic strategy employing MSCs could be added as a biologic agent or advanced therapy option (or both) used specifically to decrease inflammation, optimize wound bed preparation, and promote cutaneous healing.

## Abbreviations

14*S*,21*R*-diHDHA: 14*S*,21*R*-dihydroxydocosa-4*Z*,7*Z*,10*Z*,12*E*,16*Z*,19*Z*-hexaenoic acid
bFGF: basic fibroblast growth factor
CD: cluster of differentiation
ECM: extracellular matrix
EGF: epidermal growth factor
EMPB: *Mallotus philippinensis* bark
GPx: glutathione peroxidase
ICAM1: intercellular adhesion molecule 1
IL: interleukin
LLLT: low-level laser therapy
MMP: matrix metalloproteinase
MSC: mesenchymal stem cell/multipotential mesenchymal stromal cell
TGF-β: transforming growth factor-beta
TLR: toll-like receptor
TNF-α: tumor necrosis factor-alpha
TSG-6: tumor necrosis factor-alpha-stimulated protein 6
VEGF: vascular endothelial growth factor
